# Bioglass/Ceria Nanoparticle Hybrids for the Prophylactic Treatment of Seroma: A Comparative Short-Term Study in Rats

**DOI:** 10.1021/acsptsci.5c00327

**Published:** 2025-08-20

**Authors:** Michael-Alexander Pais, Simone de Brot, Robert Nißler, Isabel Arenas Hoyos, Athanasios Papanikolaou, Davide Bottone, Alexander Gogos, Anja Helmer, Robert Rieben, Mihai Constantinescu, Tino Matter, Inge Herrmann, Ioana Lese

**Affiliations:** 1 Department of Plastic and Hand Surgery, Inselspital, University Hospital Bern, Bern 3010, Switzerland; 2 Department for BioMedical Research, 27210 University of Bern, Bern 3008, Switzerland; 3 Department of Materials Meet Life, Swiss Federal Laboratories for Materials Science and Technology (Empa), St. Gallen 9014, Switzerland; 4 Department of Mechanical and Process Engineering, 27219 ETH Zurich, Zurich 8092, Switzerland; 5 Ingenuity Lab, University Hospital Balgrist and University of Zurich, Zurich 8008, Switzerland; 6 COMPATH, Institute of Animal Pathology, 27210 University of Bern, Bern 3012, Switzerland

**Keywords:** seroma formation, bioglass/ceria nanoparticle hybrids, animal rat model, complement protein activation, macrophage infiltration and colocalization with nanoparticles

## Abstract

Seroma formation remains a common postoperative complication. While optimal treatment remains unclear, recent attention has turned to bioglass/ceria nanoparticle (NP) treatment for seromas. Previous work showed complete seroma resolution in a rat model after NP treatment in the long term. This study aimed to assess the short-term prophylactic effects of NPs. Twenty male Lewis rats underwent bilateral seroma induction surgery. Postoperatively, seroma cavities were treated with NPs, vehicle buffer solution, or fibrin glue or left untreated. Over 2 weeks, blood, seroma fluid, and tissues were collected for biochemical, histopathological, and immunohistochemical analyses. By day 14, NP-treated seromas showed 100% fluid resolution. In contrast, seromas persisted in 50% of fibrin glue–treated rats, 60% of vehicle-treated rats, and 44.44% of untreated controls. Furthermore, prophylactic NP treatment resulted in decreased levels of inflammatory markers while the effect of fibrin glue was to increase the pro-inflammatory response. Histologically, a reduction in vascularization and individual macrophage infiltration was observed in seroma superficial capsules after NP treatment, while complement proteins were significantly increased and associated with groups of macrophages that colocalized with NPs. At the end point, NPs did not show any biodistribution to the systemic circulation. Prophylactic NP application reduced early seroma manifestations mostly through their anti-inflammatory effects. Members of the complement cascade were also identified in macrophages that colocalized with NPs and were internalized. Moreover, there were no detectable adverse systemic effects. These findings emphasize the clinical potential of NPs in the prevention of seromas and their potential for clinical use.

## Graphical abstract

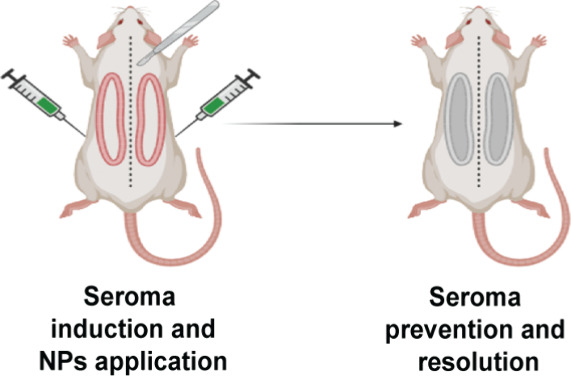

Postoperative seromas are a complication that can vary in severity, patient symptoms, and recurrence patterns, even when therapeutic interventions are carried out. Postoperative seroma formation may cause prolonged wound drainage, necessitate repetitive and frequent patient follow-ups for fluid aspiration, present an increased risk for surgical site infection, and even warrant surgical revision. Treatment may also be tedious as there is no straightforward therapeutic solution that fits all cases.^1^ In general, treatments begin conservatively with less invasive and nonsurgical approaches before escalating to surgical ones. For example, one may wait for a postoperative seroma to spontaneously resolve in cases where it is small and not causing any particular symptoms, although placement of a surgical drain or needle aspirations is common until the seroma cavity has subsided. In addition to surgical options such as radical debridement or capsule marsupialization, the application of sclerosing agents within the established seroma capsule or the potential dead space on the surgical site has also been tried, with the goal of eliminating fluid space by making the tissue planes stick together.^2–5^

Deciding whether to use this proactive approach as a preventive measure, rather than as a treatment, requires a deep understanding of the underlying mechanisms of seroma formation.

Biochemical and hematologic analyses of seroma fluid interestingly showed that its composition is different from lymph or blood serum, being more than that of inflammatory exudate.^6^ Moreover, seroma fluid seems to lack fibrinogen, thus making coagulation improbable as a mechanism of seroma cessation.^7^ Nevertheless, substitution of antifibrinolytic agents like tranexamic acid perioperatively and postoperatively demonstrated a significant reduction in the postoperative drainage volume of the surgical site.^8^ For fibrin glue, despite promising results in a rat model,^9^ clinical studies demonstrated no benefit for seroma reduction.^2^

We have previously demonstrated that zinc-doped strontium-substituted bioglass/ceria nanoparticles increased skin flap survival through neo-angiogenic and anti-inflammatory mechanisms in an animal model. Moreover, our group has demonstrated that nanoparticles (NPs) reduce seroma formation when applied therapeutically in a rat model of surgically induced seroma formation and has also recently assessed the long-term effects of NP-based seroma treatment in rats.^10–12^

An important aspect of nanoparticle biodistribution in vivo is their correlation with macrophages, as observed through electron microscopy.^13^ The complement system also seems to play a critical role in this process: upon activation by pathogens, the complement system initiates local inflammation and clearance mechanisms, including opsonization and phagocytosis.^14,15^ Nissler et al. reported that mixed metal oxide nanocatalysts, which are beneficial for wound healing and seroma treatment, were internalized by macrophages when tagged with complement factors and other markers.^13^ These findings combined indicate that nanoparticles possess anti-inflammatory properties, effectively reduce seroma formation, and promote tissue adhesions that help prevent seroma recurrence. Based on this knowledge, we aimed to further explore the prophylactic potential of NPs in a rat model over a period of 2 weeks post surgery.

## Results

### Early Seroma Volume Reduction by Prophylactic Treatment with NPs Compared to Other Short-Term Conditions

To evaluate the prophylactic effect of NP treatment on seroma volume, seromas were surgically induced in a rat model on day (D) 0. At this time point, prophylactic treatment regimens were administered based on group allocation: NPs or fibrin glue was applied on one side, while the contralateral sides received either dilution buffer solution (confined to the NP-treatment groups) or were left untreated (confined to the fibrin glue-treated groups). By postoperative day (POD) 7, bilateral postoperative seromas were observed in all rats, and each seroma was aspirated at this time point to record its volume. The use of fibrin glue treatment resulted in significantly larger seroma volumes compared to all other groups (Figure 1C). Fluid aspirates were also recorded at POD 14. Seroma fluid volumes were evaluated postaspiration as a percentage of the original recorded volume [VOLpct]. We observed a consistent reduction in fluid volumes over time in all short-term groups. However, by POD 14, seroma fluid was completely reduced on the NP-treated sides. In contrast, seromas persisted in 50% of the fibrin-treated sides, 60% of the buffer-treated sides, and 44.44% of the untreated sides. Treatment with NPs indicated the earliest and most complete volume reduction in this clinical model, with significant reductions (Figure 1) versus buffer solution treated (*p = 0.0325), fibrin-treated (*p = 0.0229), and untreated sides (*p = 0.0229) at POD 14.

**Figure 1:**
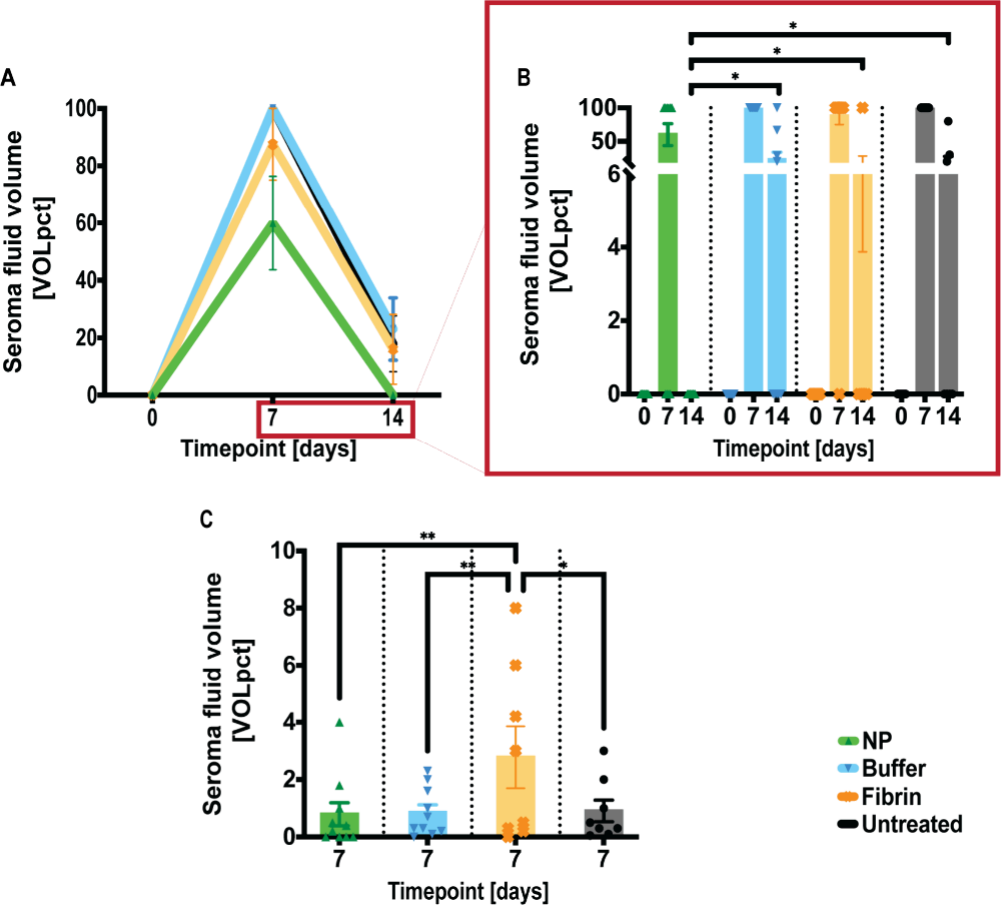
Early and complete seroma reduction with NPs compared to short-term controls. (A) After seroma induction on D0, the treatment sides were injected with either NPs or fibrin glue. At PODs 7 and 14, seroma fluid volumes were assessed after aspiration and are shown as percentages of the recorded volumes [VOLpct]. (B) NP treatment resulted in early and complete fluid reduction at PODs 14 compared to the controls. Data = mean ± standard error of the mean. *p = 0.0229, NPs vs fibrin glue and untreated, respectively, *p = 0.0325, NPs vs buffer, at POD 14. Mann–Whitney tests for single comparisons. Two-way ANOVA with Tukey posthoc for multiple comparisons indicated no significant differences between groups. (C) The largest recorded seroma volumes were registered after fibrin glue treatment compared to the controls and indicated as percentages of the recorded volumes. Data = mean ± standard error of the mean. **p = 0.0061, fibrin glue vs NPs, **p = 0.0038, fibrin glue vs buffer solution, and *p = 0.0137, fibrin glue vs untreated, respectively, at POD 7. Two-way ANOVA with Tukey posthoc for multiple comparisons.

### No Short-Term Biodistribution of NPs to the Systemic Circulation

To assess whether NPs were distributed systemically to the blood following application, blood analyses were performed using inductively coupled plasma spectroscopy (ICP-MS) to identify elemental Ce.^11^

Samples collected at defined time points (PODs 7 and 14) revealed very low cerium (Ce) levels compared to the biological baseline (D 0) of untreated whole blood and rats that had not undergone surgery, with a median of 0.47 ng/mL (Figure 2). Notably, elemental analysis showed virtually no clearance of Ce into the systemic circulation by the end point (POD 14). These findings align with minimal to no biodistribution of NPs when applied acutely to treat seroma.^10,16,17^ Furthermore, our prior long-term study using the same NPs and seroma model demonstrated the absence of cerium accumulation in blood and major organs (liver, spleen, and kidneys), reinforcing the systemic safety profile of the material.^10^

**Figure 2:**
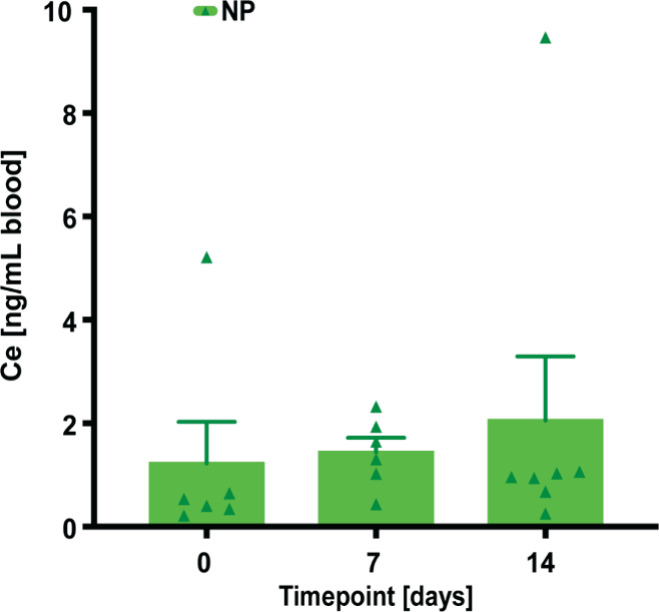
At euthanasia (POD 14), the systemic distribution of NPs was quantified by measuring cerium (Ce) levels using inductively coupled plasma mass spectrometry (ICP-MS). Initial elemental analysis of Ce in whole blood on day 0 revealed negligible levels, which remained consistent in subsequent analyses. Data = mean ± standard error of the mean. Kruskal–Wallis with Dunn post hoc for multiple comparisons indicated no significant differences between the different time points.

Plasma levels of organ-damage markers (BUN, creatinine, triglycerides, ASAT, and ALAT) were measured at specific time points to evaluate potential short-term systemic effects of NP treatment (Figure S1). As anticipated, no significant differences were observed between groups, suggesting the absence of immediate systemic responses following NP treatment.

### Inflammatory Markers: NP Treatment versus Fibrin Glue Treatment

After seroma fluid induction/prophylaxis on D 0, we explored potential plasma responses to NP treatment by quantitatively analyzing plasma analytes (such as TNF alpha, IL-1 beta, IL-2, INF gamma, and MCP-1) at designated time points (PODs 7 and 14). We noted decreased levels of inflammatory IL-1beta, IL-2, and INF gamma following NP treatment compared to fibrin glue. Conversely, levels of MCP-1, a monocyte chemoattractant, were elevated in the NP-treated group compared to the fibrin glue treatment (Figure 3).

**Figure 3:**
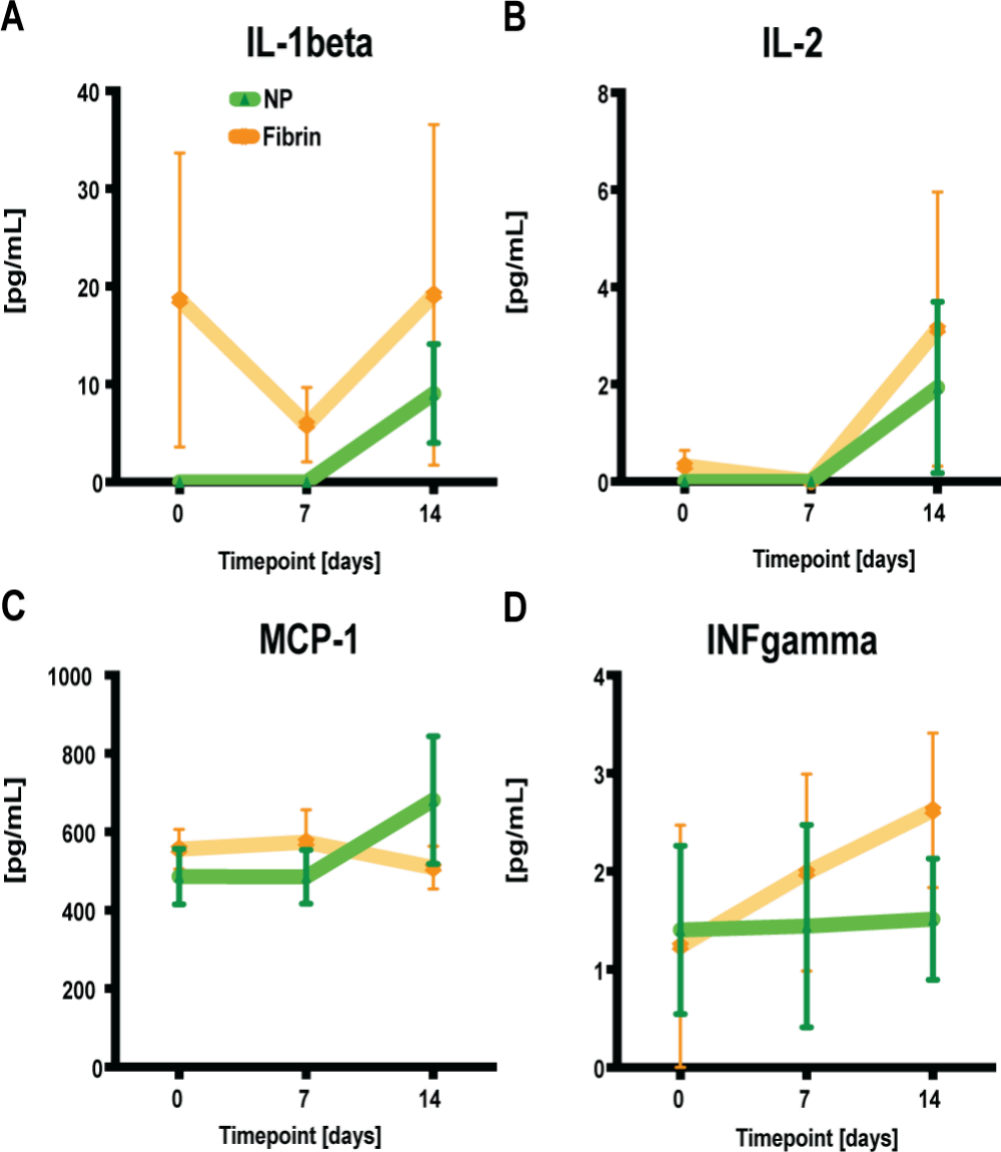
(A–D) Quantitative assessments of plasma analytes using a commercial kit (Luminex, BioPlex). When pg/mL values were not detectable (<9.45 for IL-1beta, <1.68 for IL-2, <3.27 for IFN-gamma, and <14.7 for MCP-1), they were assigned a bin value of 0. Data = mean ± standard error of the mean. Kruskal–Wallis tests with Dunn post hoc for multiple comparisons indicated no significant differences between the different treatments.

We also examined the same analytes as inflammatory-response indicators in aspirated serous fluid (Figure 4). As the NP-treated group lacked this fluid after POD 7, only a comparison between the buffer solution, fibrin glue-treated, and the untreated side was possible. The results indicated a significant increase in IL-1beta and MCP-1 in the fibrin glue-treated group at PODs 14, compared to the buffer solution-treated group (*p < 0.0158, IL-1beta at POD 14, *p < 0.0324, MCP-1 at POD 14). Already at POD 7, we could observe an increased inflammatory response after fibrin glue treatment when looking at IL-10, compared to the controls (*p < 0.0495).

**Figure 4:**
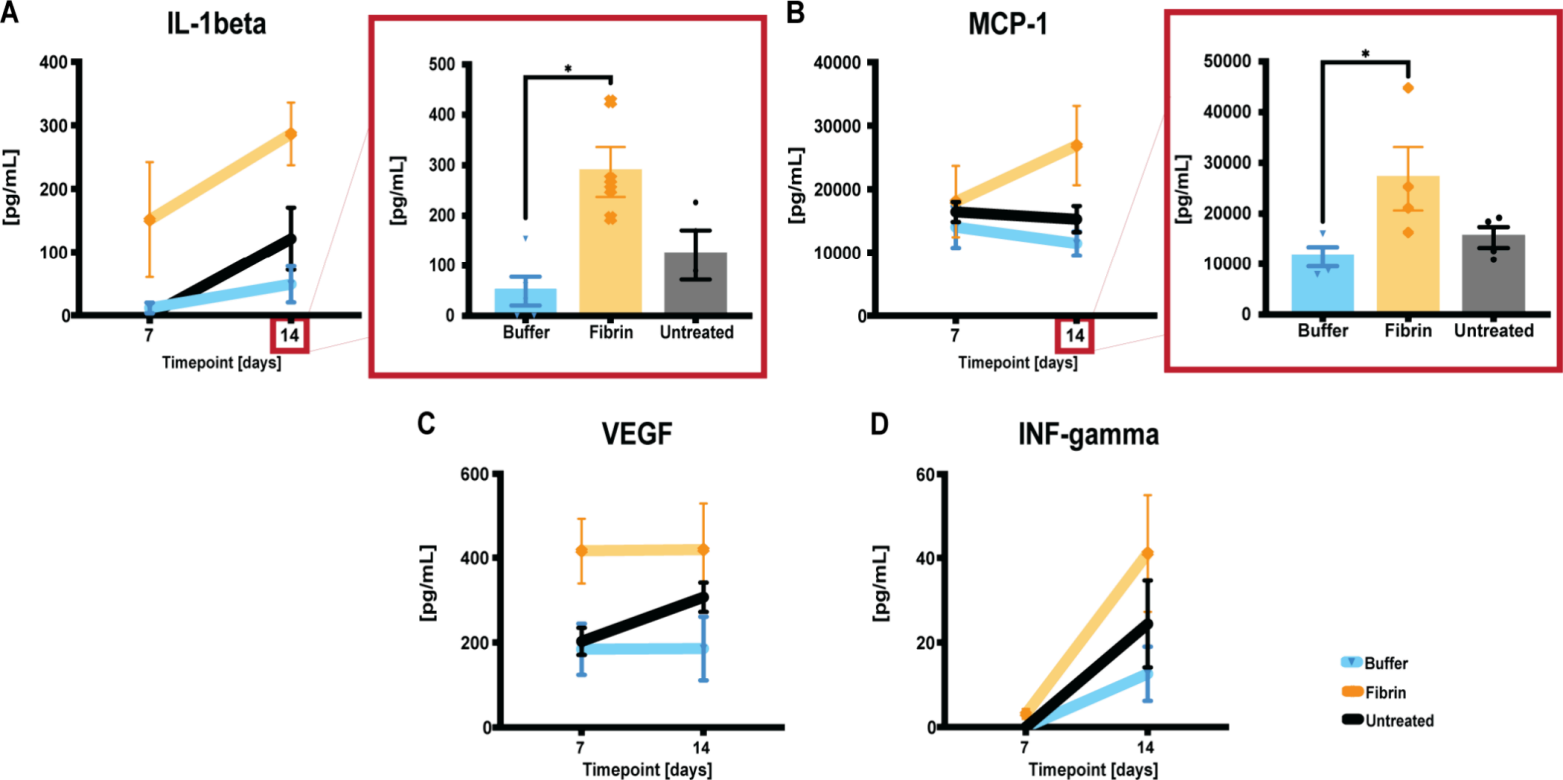
At PODs 7 and 14, quantitative assessments of seroma fluid cyto- and chemokines (A–D) were performed using a commercial kit (Luminex, BioPlex). The lack of seroma fluid in the NP-treated group after D 0 meant that only fibrin glue vs. buffer solution vs. untreated comparisons were possible. When pg/mL values were not (<5.45 for VEGF, <9.45 for IL-1beta, <6.25 or for IL-10, <3.27 for INF gamma, and <14.7 for MCP-1), they were given a bin value of 0. Data = mean ± standard error of the mean. *p < 0.0158, buffer solution vs fibrin glue, at POD 14, for IL-1beta. *p < 0.0495, buffer solution and untreated vs fibrin glue, at POD 7, for IL-10. *p < 0.0324, buffer solution vs fibrin glue, at POD 14, for MCP-1. Kruskal–Wallis tests with Dunn post hoc for multiple comparisons.

When skin tissue samples were examined along with superficial and deep seroma capsules at POD 14, levels of IL-1beta and MCP-1 analytes were lower in the NP group compared to the other groups (Figure S2 and Figure 5). However, there were no significant differences observed between the groups.

**Figure 5:**
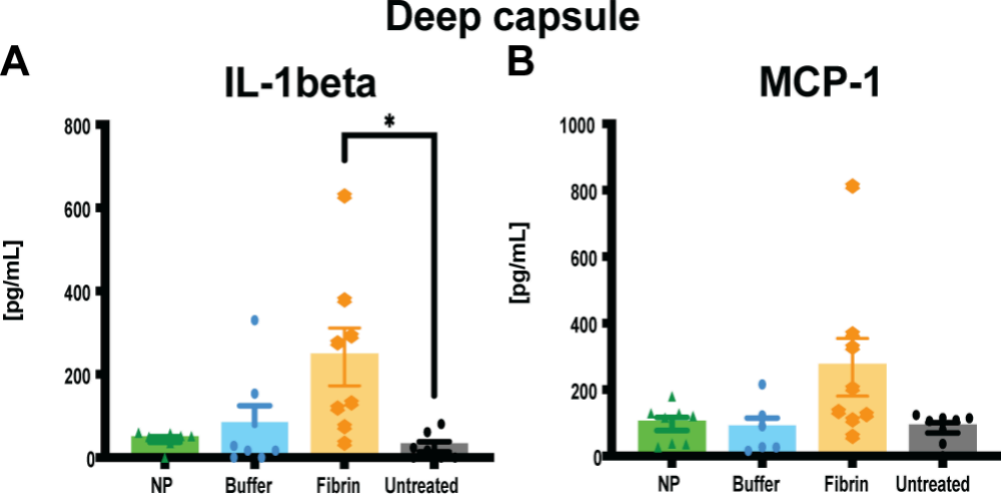
Biochemical analyses of IL-1beta and MCP-1 in deep capsule tissue (A, B) harvested at end point (POD 14). When pg/mL values were not detected (<9.45 for IL-1beta and <14.7 for MCP-1), they were given a bin value of 0. Data = mean ± standard error of the mean. *p < 0.0143, fibrin glue vs untreated, for IL-1beta. The Kruskal–Wallis tests used for MCP-1 levels from the deep capsule showed no significant differences between groups.

In contrast, the fibrin glue-treated group exhibited significantly increased levels of IL-1beta in the deep capsule compared to the untreated group (*p < 0.0143).

### Increase in Macrophage and Endothelial Cell Recruitment after Fibrin Glue Treatment as Demonstrated by Immunofluorescence

Based on principles of wound healing and peritoneal adhesion formation,^18–20^ it is reasonable to assume that seroma formation and adhesions involve early inflammatory processes characterized by neutrophil recruitment, followed by complement factor^13^ and macrophage activation and subsequent coagulation cascade activation. As demonstrated in earlier works, NP treatment favors the formation of an abundant fibrin/collagen matrix and angiogenesis, representing a fibrin–fibrinolysis imbalance.^19^ In past studies, NP treatment would eventually lead to adhesion formation, primarily marked by late-stage macrophage infiltration and depositions of extracellular substances, presumably collagen.^10,19^

We therefore decided to use immunofluorescence (IF) to label crucial cell markers and structural proteins during the various stages of seroma formation. Figure S3 displays IF staining of skin tissue cross sections following NP, buffer solution, and fibrin glue treatments, as well as the untreated side. Our analyses focused on the following markers: CD68 (for macrophages), CD31 (for endothelial cells of vessels), and blue DAPI representing cell nuclei (Figure 6 and Figure S3).

**Figure 6:**
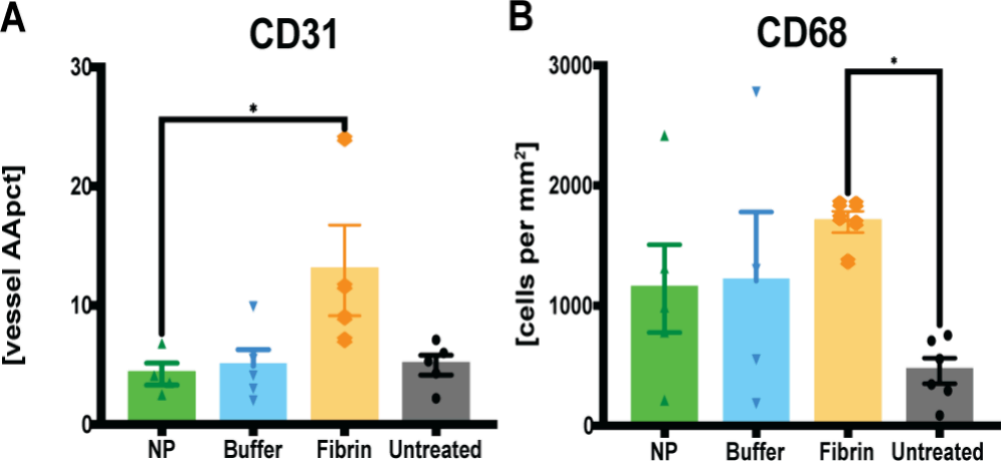
(A) Decreased vascularization after NP treatment. Immunofluorescence staining for CD31, determining CD31-positive vessel count, indicated as vessel density [VD]. Data = mean ± standard error of the mean. *p = 0.0286, NPs vs fibrin glue, Mann–Whitney tests for single comparisons. Kruskal–Wallis tests with Dunn post hoc for multiple comparisons with no significant differences between the different treatments. (B) Decreased individual macrophages after NP treatment, and increased macrophage infiltration after fibrin glue treatment, indicated as cell counts [per mm^2^] for CD68. Data = mean ± standard error of the mean. *p = 0.0235, untreated vs fibrin glue. Kruskal–Wallis tests with Dunn post hoc for multiple comparisons.

Our findings revealed a smaller increase in labeled endothelial cells, identified by CD31 (*p = 0.0286), and individual macrophages, identified by CD68, following NP treatment compared to fibrin glue treatment, particularly near the superficial capsule (Figure 6). CD68 was significantly increased especially after fibrin glue treatment (*p = 0.0235) (Figure 6). It is well-known that endothelial cells and macrophages are essential for wound healing and maturation, regulated by different stages of inflammation, skin wound angiogenesis, and infiltration.^18,20^ Based on our previous findings that NPs exhibit anti-inflammatory properties, our current results reinforce this by demonstrating reduced vascularization following NP treatment, while also highlighting the detrimental pro-inflammatory effects of fibrin glue.^12^

### Heightened Complement Factor (C3c) Depositions after NP Treatment as Demonstrated by Immunohistochemistry

Relevant features of seroma formation, especially macrophage and complement factor infiltration, vascularization, and deposition of COL1, were evaluated using immunohistochemistry (IHC) analyses of the skin and superficial capsule tissue run after euthanasia (EP/POD 14) (Figure 7). Tissues, i.e., skin/superficial capsule, were harvested, processed, and analyzed further using IHC. We specifically examined the levels of infiltration of complement factors (C3c), individual macrophages (CD68), vascularization (CD31), and COL1 depositions (Figure 8).

**Figure 7:**
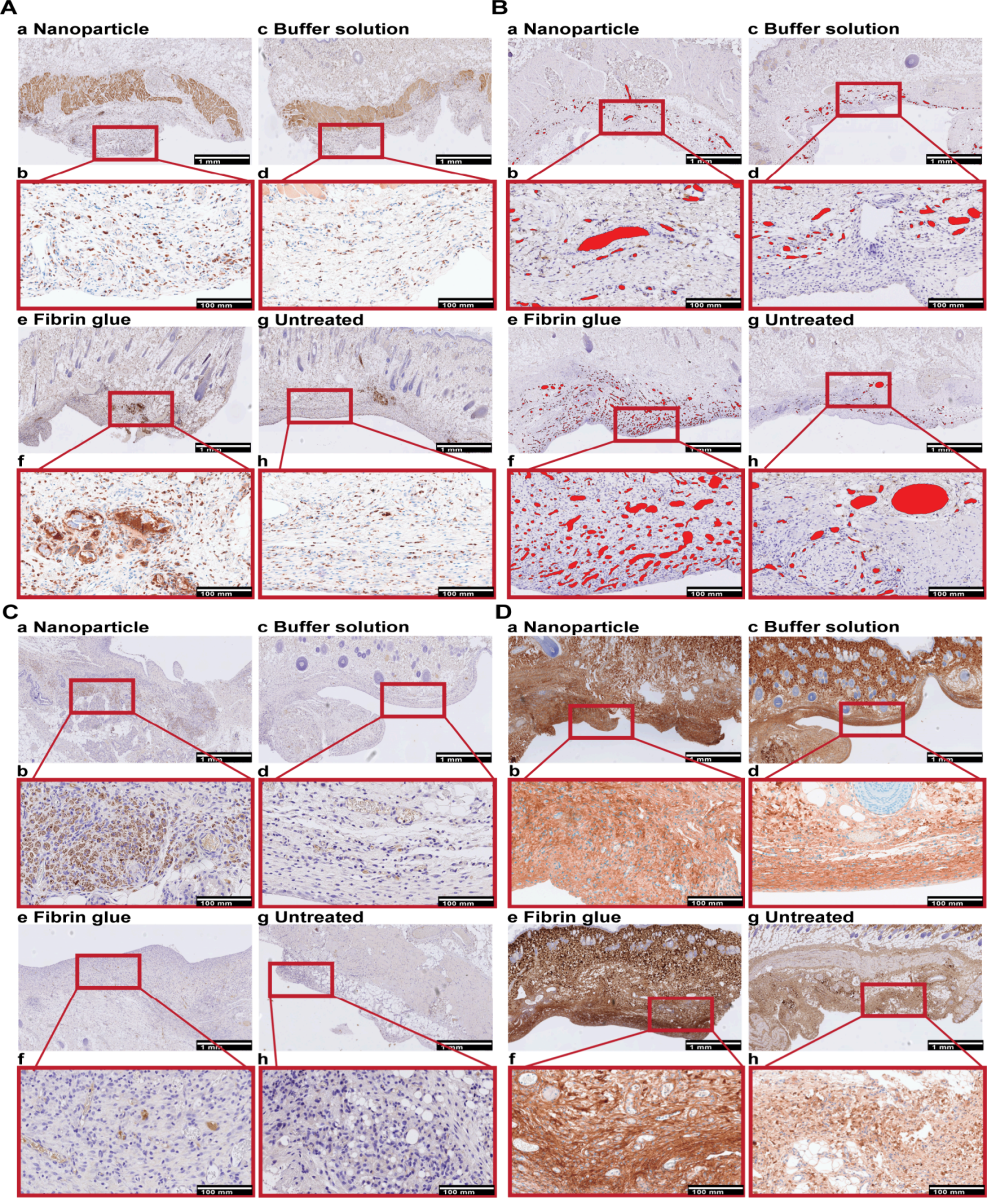
Immunohistochemical (IHC) staining for macrophages (CD68), complement factor C3c, vessels (CD31), and collagen type 1 (COL1) in skin containing the superficial seroma capsule of NP-treated (a, b), buffer-treated (c, d), fibrin glue-treated (e, f), and untreated groups (g, h). Overview and enlarged areas at the capsular level of skin tissue sections. Scale bars, 1 and 100 mm. (A) CD68. Individual cells of CD68-positive cells are less present after NP treatment, but most extensive after fibrin glue treatment. (B) C3c. Individual cells of C3c-positive cells are most extensive after NP treatment, with macrophage-oriented depositions. (C) CD31. CD31-positive vessels are labeled in red. The level of vascularization is lower in NP compared to other treatment groups. (D) COL1. Collagen deposition tended to be multifocal and to be denser after NP treatment compared to other treatment groups.

**Figure 8:**
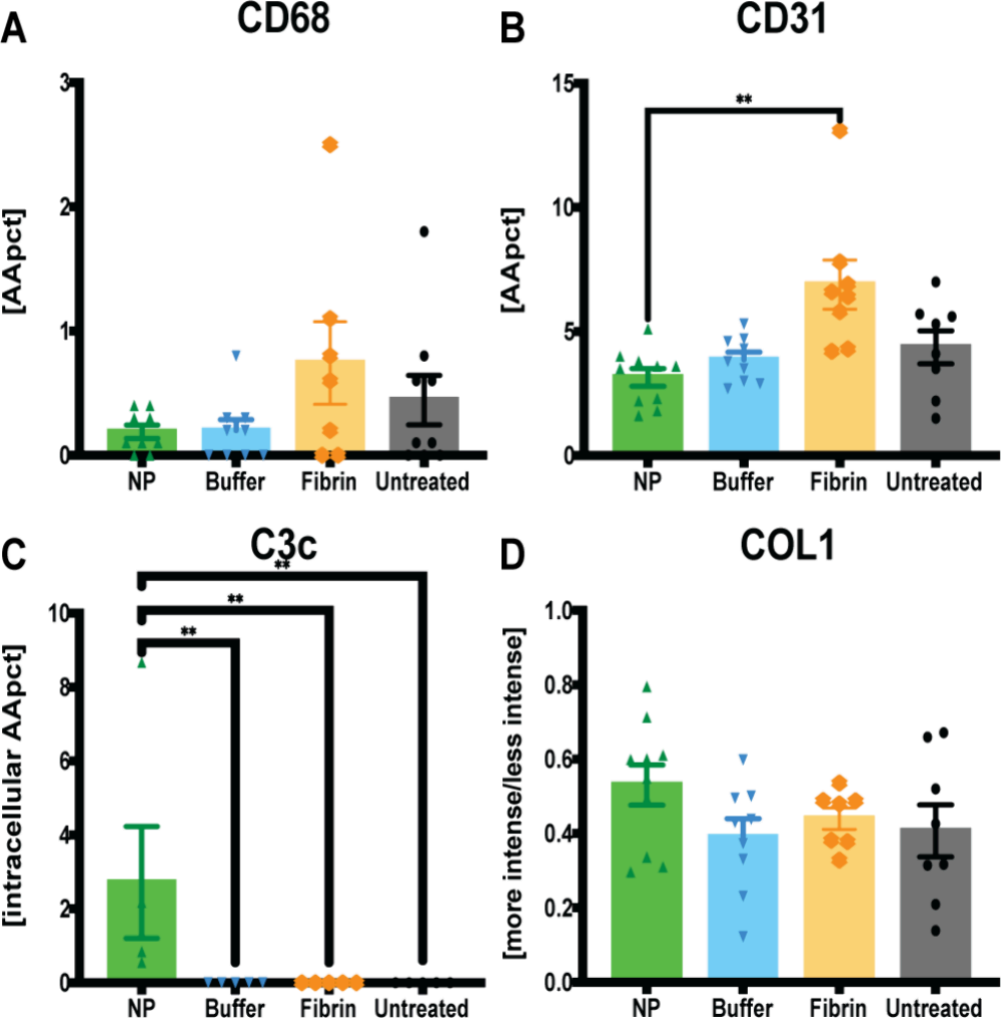
Quantitative assessments of immunohistochemistry staining for macrophages (CD68), complement factor C3c, endothelial cells (CD31), and collagen type 1 (COL1) in capsule tissue, comparing four treatment conditions: NP vs. buffer vs. fibrin glue vs. untreated. (A) The least individual macrophage infiltrations, indicated as percentage of the assessed capsule tissue [AApct], were observed after NP treatment. Data = mean ± standard error of the mean. One-way ANOVA with Tukey post hoc for multiple comparisons with no statistical significance. (B) Decreased vascularization after NP treatment. IHC staining for CD31, determining CD31-positive vessel count, indicated as a percentage of the assessed capsule tissue [AApct]. Data = mean ± standard error of the mean. **p = 0.0015, NP vs fibrin glue. Kruskal–Wallis tests with Dunn post hoc for multiple comparisons. (C) Increased individual C3c infiltrations, indicated as percentage of the assessed capsule tissue [AApct], were observed after buffer solution and in the untreated side. Data = mean ± standard error of the mean. **p = 0.0027, NPs vs fibrin glue, vs buffer solution, and vs untreated, respectively. One-way ANOVA with Tukey post hoc for multiple comparisons. (D) Increases in more intense collagen type 1 deposition after NP treatment. IHC staining for COL1 measuring the ratio between more vs less intensely stained (et al., densely arranged) collagen, [more intensity/less intensity]. Data = mean ± standard error of the mean. One-way ANOVA with Tukey post hoc for multiple comparisons with no statistical significance.

Intracellular C3c was exclusively detected in NP samples, with heightened signaling observed in macrophages located near the superficial capsule. Quantitative analysis revealed a significant difference when compared to all control groups (**p = 0.0027). IHC analysis also showed significantly reduced CD31 signaling after NP treatment, with the highest levels seen in the fibrin-treated group (**p = 0.0015). The fibrin-glue-treated group also exhibited significantly increased macrophage infiltration, identified by CD68, compared to both the buffer solution (**p = 0.0011) and untreated groups (*p = 0.0185). In contrast, NP-treated samples displayed fewer macrophage infiltrations, although the difference was not statistically significant when compared to fibrin glue treatment. Interestingly, granulomas with presence of intracellular material interpreted as NP were only observed in NP samples. This may be due to the fact that, despite their anti-inflammatory properties, NPs could aggregate over time, leading to uptake and retention within macrophages. Denser deposits of COL1 were noted within the seroma capsule tissue following NP treatment, in contrast to the control group (Figure 8). For COL1 density analysis, we compared the ratios of looser to denser collagen areas and vice versa.

### Colocalization of Complement Factors (C3c) alongside Macrophages with NPs Using Correlative Microscopy

As a next step, when digitally aligning IHC and scanning electron microscopy (SEM) analyses, we confirmed colocalization of a complement protein (C3c) within individual macrophage staining and NPs, indicating a role for the complement system in macrophage recruitment and their responsibility for the uptake and retention of NPs at the site of application (Figure 9).

**Figure 9:**
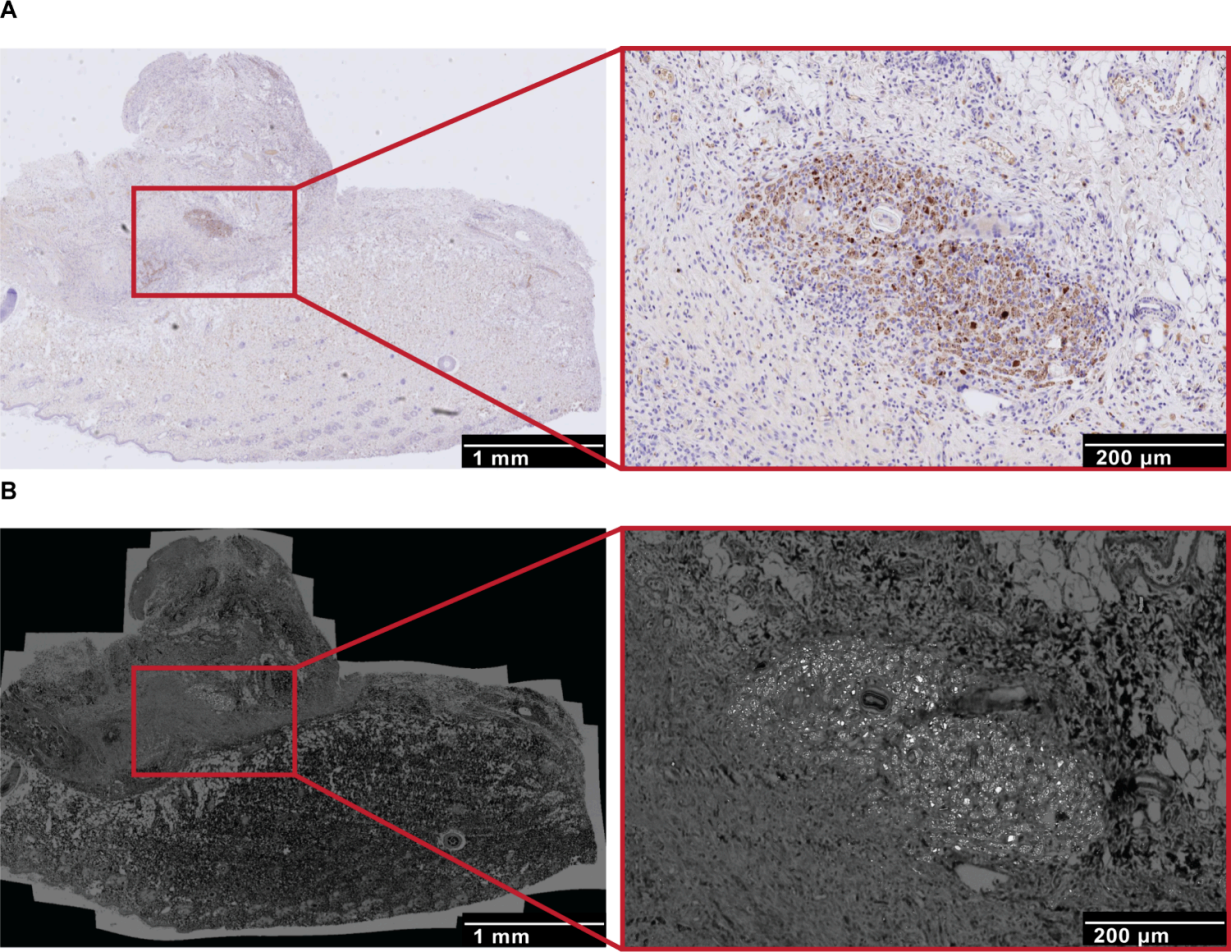
Correlation between IHC and SEM imaging techniques. (A) Immunohistochemical staining of complement factors (C3c) in macrophages within the capsule tissue of nanoparticle (NP)-treated groups. The image shows focal granulomas with C3c-positive complement staining and CD68-positive macrophages containing intracellular purple-gray material, indicative of NP accumulation. Scale bar: 1 mm. Inset: higher magnification, scale bar: 200 μm. (B) Corresponding SEM images, collected using a backscattered electron detector, highlighting regions from the immunohistochemical analysis (scale bars: 1 mm and, at higher magnification, 200 μm). Ce-containing NPs appear as bright spots.

## Discussion

These findings underscore the effectiveness of NPs in averting seroma formation within an established rat model, administered as a prophylactic treatment regimen, prior to seroma onset. In our previous studies, we demonstrated the therapeutic impact of these NPs, not only significantly mitigating early signs of seroma but also inducing an anti-inflammatory response and fostering increased adhesion formation over the long term. This suggested a reduced risk of seroma recurrence.^10,12^ While those long-term investigations support the utilization of bioactive inorganic nanoparticles for the safe and efficient therapeutic management of seroma, little was understood regarding their prophylactic potential in preventing seroma formation.

Our current findings indicate that the preemptive application of NPs have a prophylactic effect on early seroma formation, resulting in complete seroma volume reduction by POD 14 compared to control groups. This effect appears to be predominantly driven by the anti-inflammatory properties of NPs, as evidenced immunohistochemically by reduced macrophage infiltrations and vascularization during the acute phase of seroma formation. Notably, these results stand in contrast to those associated with fibrin glue, which displays pro-inflammatory characteristics and seems less effective in minimizing postoperative seroma formation. Analyses of inflammatory markers from samples of aspirated serous fluid and deep seroma capsule tissue revealed a significantly increased inflammatory response after fibrin glue treatment compared to the controls. Similar observations of a pro-inflammatory response following fibrin sealant application were made when examining the seroma capsule immunofluorescently and histochemically. Despite its frequent use in surgical practice for tissue adhesion and dead space elimination, fibrin glue has not been definitively established as an effective treatment for seromas.^1,2,9,21,22^ The observed pro-inflammatory component, manifested by increased numbers of macrophages and endothelial cells, may explain the ineffectiveness of fibrin glue in clinical settings.^10,12^

The beneficial use of NPs within a clinical context is further supported by their lack of systemic response, as evidenced by the absence of significant differences in NP biodistribution in the bloodstream following euthanasia, as demonstrated by ICP-MS, after early prophylactic application of these hybrid NP formulations. This implies that the administration of NPs does not lead to notable dysfunction in other organ systems.

Conversely, within our current study, the treatment with fibrin sealant produced contrasting results. Animals treated with fibrin glue not only exhibited persistent seromas but also appeared more susceptible to dehiscences and hematoma formation. These findings align with previous research indicating adverse reactions associated with fibrin glue use, such as a heightened bleeding risk and sustained seroma formation.^23–25^

While complete safety in a clinical setting cannot be definitively established, our findings strongly suggest an absence of significant systemic toxicity in this model.

Despite an unclear understanding of seroma pathogenesis, our research has identified potential pivotal mechanisms underlying its formation. In our long-term study, microscopic examination revealed late-stage macrophage infiltration and collagen type 1 depositions within the superficial capsule. This highlights the crucial role of macrophages and COL1 in adhesion formation and reducing the risk of seroma recurrence. Moreover, macrophages were found to colocalize with NPs, suggesting NP uptake and retention at the treatment site.^10^

During this study, the prophylactic treatment of seromas with NPs provided clearer physiopathological insights into seroma formation. It became necessary to distinguish between the early and late stages of seroma development. In the acute phase, endothelial cells and individual macrophages appeared to drive inflammation and seroma development. However, as time progressed, particularly following NP application, late-stage macrophage infiltration, and their aggregation into clusters (granulomas), along with the deposition of collagen type 1, emerged as critical factors influencing NP uptake and retention. Ultimately, these processes contributed to the resolution of the seromas.^10,13^

Remarkably, our findings indicate that the complement system, notably C3c, might play a pivotal role in the early stages of seroma formation and could be activated by NPs. Through immunohistochemical analyses, we observed a distinct accumulation of C3c with clustered macrophages, which subsequently demonstrated colocalization with NPs, as confirmed by SEM analyses. This underscores the significance of the interaction between early C3c activation, late-stage macrophage infiltration, and NPs. Similar observations were made by Nissler et al.^13^ who demonstrated that increased complement factors and other opsonins on nanocatalyst surfaces facilitated their uptake into macrophages when applied topically.

Considering that complement factors are widely recognized for their pivotal role in the wound repair process, particularly in inflammation, they facilitate the recruitment and subsequent differentiation of macrophages, which are essential for tissue formation and remodeling.^20^

## Limitations

Here, we emphasize the significance of early-stage complement factor C3c, macrophage infiltration (CD68), and COL1 deposition following NP treatment, which may contribute to seroma reduction and cavity closure. However, the precise pathways governing seroma formation and resolution remain complex. Therefore, future molecular studies focusing on the pathophysiology of seroma formation and the mechanism of action of NPs are essential.

The observed trend toward anti-inflammatory actions by NPs, as evidenced by their effects on plasma fluid, seroma aspirate, and tissue qualities, is both descriptive and biochemically quantifiable. While control cases only treated with buffer solution displayed similar biochemical behavior,^26^ it was only in cases treated with NPs that a complete remission in effect was observed, demonstrating a beneficial preventive use. The formulation of the vehicle buffer solution warrants re-examination and potential revision since the combined application of NPs and buffer solution seems to have a strong sclerosant effect for seroma cavity closure.

Lastly, the development of a large animal model for seroma formation is critical for preclinical testing. Utilizing such a model to compare NPs to a gold standard like fibrin glue would further validate the clinical value and effectiveness of bioactive inorganic NPs.

## Conclusions

Our study has not only highlighted the complexity of bioglass/ceria hybrid nanomaterials and their ability to interact with various proteins, such as complement factor C3c, macrophages, and collagens, but it has also provided insights into potential pathogenesis patterns and the intricacies of seromas. Our findings underscore the promising potential of NPs for clinical prophylaxis and therapy of seromas.

## Materials and Methods

### Nanoparticle Synthesis

Zinc-doped and strontium-substituted bioglass/ceria nanoparticles were synthesized using flame spray pyrolysis, following established procedures outlined in prior literature.^11,12,27^ Initially, a mixture of calcium 2-ethylhexanoate, sodium 2-ethylhexanoate, tributyl phosphate, hexamethyldisiloxane, strontium acetylacetonate hydrate, Ce-2-ethylhexanoate, and zinc acetylacetonate in tetrahydrofuran (THF) was prepared and sprayed through a nozzle. Upon ignition, the droplets underwent nucleation, resulting in the formation of finely dispersed nanoparticles collected in powder form. These nanoparticles were then dispersed in modified Ringer’s lactate buffer, supplemented with citric acid and sodium citrate at 7.5 mM each.^16,28^ Stored in Eppendorf tubes at room temperature prior to dispersion, the nanoparticles are readily available for immediate utilization in various biomedical applications, as highlighted in previous studies.^11,17^

### Nanoparticle Characterization

The synthesized nanoparticles underwent analysis via X-ray diffraction (XRD) using a Bruker D2 s Gen Phaser with Cu Kα radiation at 2θ = 10–80° and a step size of 0.03°, operating at 40 kV and 40 mA. Phase composition was determined utilizing Diffrac.EVA (V3.1) software.^16^ Surface area assessment was conducted using the Brunauer–Emmett–Teller (BET) method at 77 K with a Micromeritics TriStar II Plus instrument.^27^ Elemental composition analysis was performed following the optimized nanoparticle digestion protocol outlined in Section 2.5, via inductively coupled plasma optical emission spectroscopy (ICP-OES) using an Agilent instrument based in Santa Clara, California.^8,9^

### Surgical Model of Seroma Induction

Bilateral seroma induction surgeries were performed as described previously in our rat model: An incision was made along the posterior axillary line, the cutaneous maximus and latissimus dorsi muscles were excised, and a partial axillary lymphadenectomy was performed.^12^ Additionally, the undersurface of the skin flap was scraped with a scalpel. Skin closure was performed with Vicryl 4-O sutures (coated VICRYL polyglactin 910, Ethicon US, LLC).

All surgical and interventional procedures in rats were performed using balanced anesthesia: fentanyl 0.0005 mg/kg, medetomidine 0.15 mg/kg, and midazolam 2 mg/kg, administered subcutaneously (s.c.). After administering this anesthetic cocktail, oxygen (O_2_) was administered until loss of consciousness. During surgery, 100% O_2_ was used. In cases of prolonged anesthesia, inhalational maintenance anesthesia was administered using 1–2% isoflurane. Rats were maintained at normal body temperature using thermal pads, and ophthalmic ointment was applied to the eyes. Continuous monitoring (respiratory rate, temperature) was provided until awake. At the end of surgery, anesthesia was reversed with a cocktail containing buprenorphine 0.05 mg/kg, atipamezole 0.75 mg/kg, and flumazenil 0.2 mg/kg s. c. For continuous analgesia, meloxicam 1 mg/kg was administered postoperatively. During recovery from anesthesia, rats were kept warm using a heating pad for at least 1 h. Analgesia was continued for 48 h postoperatively in drinking water (360 mL) containing both buprenorphine 0.3 mg/mL and 10 mL 5% glucose, and with meloxicam 1 mg/kg for 4 days. Rescue analgesia with buprenorphine was given when rats showed signs of additional pain, according to score-sheet assessments.^12^

Soft critical-care feed (EmerAid Omnivore, EmerAid, LLC, Cornell, Illinois, USA) was provided to support feeding and recovery for the first five postoperative days.

For weekly blood sampling and seroma aspiration, isoflurane (5% with 100% O_2_, 1 L/min) was used for initial anesthesia in an induction chamber. Maintenance anesthesia was provided using 2–2.5% isoflurane with 0.6% L/min O_2_.

For euthanasia, rats underwent terminal anesthesia using 150 mg/kg pentobarbital administered intraperitoneally (i.p.) (Esconarkon, 300 mg/mL; dilution 1:10; Streuli Pharma, Uznach, Switzerland).

This study was conducted according to ARRIVE guidelines^29^ and was approved by the Cantonal Animal Ethics Committee for Animal Experimentation, Bern, Switzerland (approval number BE 110/2020).

### Treatment Group Allocation and Experimental Design Setup

The study involved a total of 20 inbred male Lewis rats (weight range: 200–250 g), allocated into two experimental treatment groups (*n* = 10 per group). In the first group (NPs), seromas were surgically induced on D 0, and NPs were applied to one side of each animal. The contralateral side served as an internal control and received vehicle buffer solution only. In the second group (fibrin glue group), fibrin sealant (Tisseel, Baxter AG, Opfikon, Switzerland) was applied to one side of each rat, with the opposing side left untreated. This within-animal paired design enabled direct comparisons (NP vs buffer and fibrin glue vs untreated) while reducing biological variability and supporting ethical refinement through reduced animal use.

In accordance with the ethical principle of the 3Rs—Replacement, Reduction, and Refinement—the number of animals was minimized while ensuring sufficient biological replicates for both within- and between-group comparisons. All animals were housed and treated under identical conditions, with randomization of left/right treatment sides to avoid lateral bias. Between-animal comparisons (e.g., NP vs fibrin glue, NP vs untreated, buffer vs untreated, fibrin glue vs buffer) were also performed using appropriately segregated groups.

The NPs were delivered to the surgical site via a 1 mL plastic syringe equipped with a fine spray nozzle, using the same method for vehicle buffer administration. Fibrin glue was applied directly and evenly across the wound area. In all animals, skin closure was performed using Vicryl 4–0 sutures (Ethicon US, LLC), ensuring the retention of applied materials in situ.

The study was conducted as a pilot investigation to evaluate the feasibility and biological effects of the NP treatment.

The NP suspension was prepared at a concentration of 5 mg/mL in buffer solution, based on preliminary optimization experiments testing concentrations from 1 to 15 mg/mL, in which 5 mg/mL yielded the most promising initial results. A total volume of 1 mL was used for each treatment condition, including fibrin glue and buffer-only controls.

At designated time points, seroma fluid (if present) was aspirated, and 0.5 mL of blood was collected under anesthesia for biochemical analyses. At the study end point (POD 14), animals were euthanized, and tissue and organ samples were harvested for further histological and molecular evaluations (Figure 10B).

**Figure 10:**
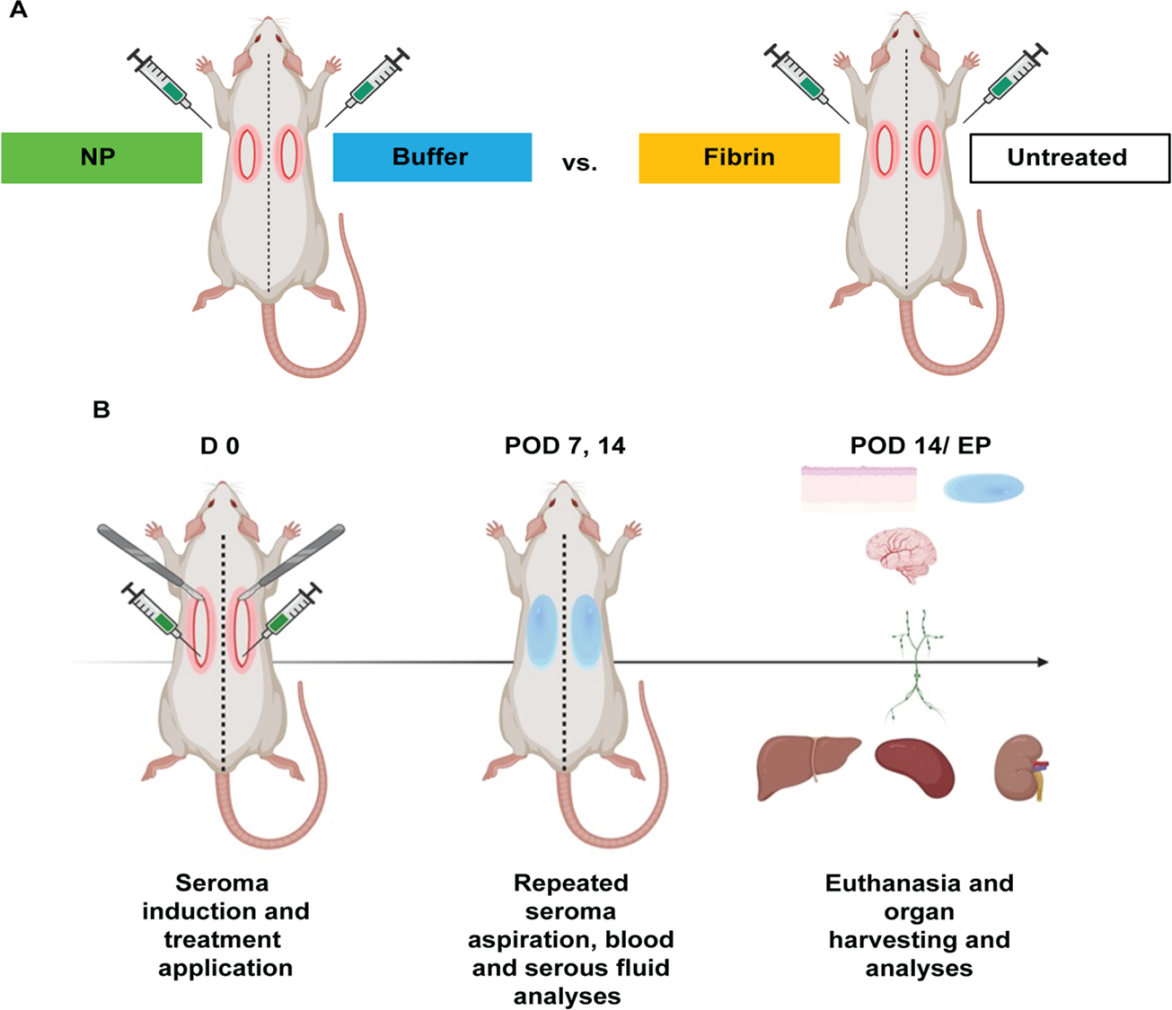
Experimental design for seroma induction, formation, aspiration, and treatments in Lewis rats (*n* = 20). (A) Seromas were surgically induced bilaterally in the axillary area of 20 Lewis rats (D 0), followed by application of BG/ceria NPs in 10 rats and fibrin glue treatment in 10 rats. Control sides were treated with either buffer solution or left untreated. (B) Seroma fluid, blood, and tissue samples were taken at defined time points. At euthanasia (POD 14/EP), NP systemic effects were assessed by blood analyses, and organ samples were assessed using biochemical, histopathological, and immunohistochemical methods.

### Elemental Analysis of Cerium (Ce) from the Whole Blood

The distribution of NPs within the blood was investigated by analyzing cerium (Ce) levels through ICP-MS.^17^ Samples of whole blood (100 μL) were transferred in a polytetrafluoroethylene digestion tube and mixed with 3 mL of 65% HNO_3_ p.a. (Merck) and 1 mL of 30% H_2_O_2_ p.a. (Merck). These samples underwent digestion using a microwave (turboWAVE, MLS GmbH, Germany) at 250 °C and 120 bar for 18 min. Subsequently, cerium levels were measured using ICP-MS (Model 7900, Agilent, Santa Clara, California) by quantifying the isotope Ce140, with Lu175 utilized as an internal standard to correct for nonspectral interference.^12,27^

### Systemic Effects of NP Treatment

Blood plasma samples collected at days 0, 7, and 14 were subjected to quantitative analysis for blood urea nitrogen (BUN), creatinine, triglycerides, alanine aminotransferase (ALAT), and aspartate aminotransferase (ASAT). These analytes are indicative biomarkers for organ failure^16^ and were assessed utilizing the Roche cobas 8000 c system (Roche Diagnostics GmbH, Mannheim, Germany).

### Cytokine Measurements

Protein extractions from blood plasma, serous fluid, and skin tissue were conducted following previously described methods.^11,12,30^ Blood and serous fluid samples were collected at predetermined time points (PODs 7 and 14). The samples underwent centrifugation twice for blood at 1000 rcf for 10 min, followed by 1500 rcf for 15 min, and once for serous fluid at 1500 rcf for 15 min.^30^ Supernatants were collected and subjected to further quantitative assessments. Skin samples weighing between 20 and 50 mg were weighed on an analytical scale, cut into small pieces, and transferred to 2 mL FastPrep Lysing Matrix tubes D containing 1.4 mm ceramic spheres (MP Biomedicals, Germany). These samples were kept on dry ice until lysate buffer addition. The lysate buffer comprised 1× protease inhibitor cocktail (Halt Phosphatase Inhibitor Cocktail, Pierce, Rockford, Illinois, USA) and RIPA buffer (Pierce, Rockford, Illinois, USA). For instance, for a 225 mg tissue sample, 2250 μL RIPA buffer, and 22.5 μL protease inhibitor were used. The tissue was homogenized using the FastPrep-24 system (MP Biomedicals, Germany) followed by centrifugation at 13,000 rcf for 1 min at 4 °C. Each supernatant was then transferred to a new tube for further analyses.

Quantitative assessments of plasma, serous fluid, and skin tissue analytes were carried out using a customized commercial kit, Rat ProcartaPlex Mix & Match 9-Plex (Thermo Scientific, Bender MedSystems GmbH, Vienna, Austria) at defined time points for the following proteins: vascular endothelial growth factor A (VEGF-A), tumor necrosis factor alpha (TNF alpha), interleukin 1 beta (IL-1 beta), interleukin 2 (IL-2), interleukin 6 (IL-6), interleukin 10 (IL-10), monocyte chemoattractant protein-1 (MCP-1/CCL2), and interferon gamma (IFN gamma). TNF alpha, IL-1 beta, IL-2, MCP-1, and IFN-γ are known for their pro-inflammatory activity, while IL-10 is an anti-inflammatory cytokine. IL-6 functions as both a pro-inflammatory and anti-inflammatory cytokine (reference 42). VEGF-A is a potent angiogenic cytokine that stimulates endothelial cell proliferation and plays a critical role in angio- and vasculogenesis. It is regulated by all stages of the wound healing process, including inflammation.^12,20,31,32^ MCP-1, a monocyte chemoattractant, and IFN gamma, a primary recruiter and activator of macrophages, are also recognized for their role in the inflammatory response.^18^ The procedure adhered to the original manufacturer’s instruction manual, and plates were read on a FLEXMAP 3D system (Luminex, Austin, California, USA).

### Histological Analysis

At the time of euthanasia (POD 14/EP), skin tissue was collected for microscopic analysis. Samples underwent rinsing with PBS, drying, and embedding in the OCT matrix (Tissue-Tek, Sakura Finetek Europe BV, Leiden, The Netherlands) and then preserved at −25 °C until they were sectioned (5 μm) using a cryostat. Other samples were rinsed in PBS, dried, and then fixed in formaldehyde for subsequent histological processing.

IF was conducted on cryosections from skin tissue samples embedded in OCT, including the superficial capsule, following the protocols described by Zhang et al.^33^ The sections were fixed with acetone and subjected to primary staining using anti-CD31/PECAM-1 (dilution 1:100; AF3628-SP, Bio-Techne), along with each of the following antibodies: anti-CD68 (dilution 1:200; ab125212, Abcam), anti-MPO (dilution 1:250; A039829, Dako), anti-C3c (dilution 1:4000; A0062, Dako), and anti-fibrinogen (dilution 1:100; A0080, Dako). Subsequently, suitable secondary antibodies [Alexa Fluor 568-labeled antirabbit IgG (A10042, 2306809, Invitrogen) and Alexa Fluor 488-labeled antigoat IgG (A11055, 552222, Molecular Probes)] were applied for imaging using a Zeiss LSM980 confocal microscope.

For histological assessments, longitudinal sections of fixed skin were trimmed, processed conventionally, and stained with hematoxylin and eosin (H&E). A blinded microscopic analysis was then conducted by a board-certified veterinary pathologist (SdB). The slides were digitally scanned using a NanoZoomer S360 Hamamatsu scanner and reviewed using NDP.view2 Hamamatsu viewing software. Relevant histopathological features were identified, and specific parameters, i.e., fibrosis, edema, vascularization, inflammation, and the presence of foreign materials (including intracellular substance indicative of NP accumulations), were semiquantitatively evaluated.

Quantitative analysis of IHC was performed digitally using Visiopharm software (Visiopharm, Horsholm, Denmark, version 2023.09 x64). All available capsule tissue was defined as the region of interest (ROI) and subjected to complete quantification of the different markers. CD68- and C3c-positive cells, and CD31 positive vessels were detected and quantified through automated deep learning classification (U-Net), which was trained and designed separately for each marker. CD68-positive granulomas were manually delineated and measured in terms of area (mm^2^). Areas exhibiting COL1 positivity were quantified using a threshold classification method, considering both the presence and intensity of staining (categorized as dense or less dense COL1 deposition).

### Scanning Electron Microscopy (SEM) Imaging

Histological specimens derived from consecutive formalin-fixed paraffin-embedded tissue sections of skin and the superficial capsule were stained for C3c protein and underwent analysis using SEM along with energy-dispersive X-ray spectroscopy (EDX) for elemental identification. To prepare for SEM, coverslips on the histological slides were removed over a 3-day incubation period in xylene. After coverslip removal, samples were air-dried and coated with a 10 nm carbon layer before imaging with an Axia ChemiSEM (Thermo Fisher Scientific) equipped with a backscattered electron detector (BSE Detector). Cerium-containing particles were identified using a combination of the BSE Detector for mass contrast and EDX spectroscopy employing a TrueSight LX Detector (Thermo Fisher Scientific).

Whole-specimen imaging was accomplished by acquiring tiled images and stitching them using the Maps software (Maps 3.18, Thermo Fisher Scientific). The stitched images were denoised with the nonlocal means method in the scikit-image implementation (v0.24),^34^ and initial registration with the IHC images was performed with the SimpleITK library (v2.4.0).^35^

### Statistical Analysis

Statistical analysis was conducted using GraphPad Prism, version 9.5.1 (GraphPad Software, San Diego, California, USA). All values are presented as mean ± standard error. Data distributions were first assessed using the D’Agostino-Pearson and Shapiro–Wilk tests. For normally distributed data, comparisons between different groups were carried out using two-way ANOVA tests with Tukey post hoc analysis for multiple comparisons. In cases of non-normally distributed data, the Wilcoxon signed-rank test was utilized for comparisons within the same group, while the Mann–Whitney *U* test was employed for single comparisons, and the Kruskal–Wallis test, followed by Dunn’s post hoc analysis, was used for multiple comparisons between groups. A significance level of *p* < 0.05 was considered statistically significant.

## Supplementary Material



## Notes

Plasma levels of organ-damage markers: BUN, creatinine, triglycerides, ASAT, and ALAT (Figure S1); biochemical analyses of IL-1β and MCP-1 in skin/superficial capsule tissue (Figure S2); and confocal microscopy of immunofluorescent staining for macrophages (CD68), endothelial cells (CD31), and nuclei (DAPI) in skin/capsule tissue (Figure S3) (PDF)

## Abbreviations

ALAT: alanine aminotransferase
ASAT: aspartate aminotransferase
BET: Brunauer–Emmett–Teller method
BUN: blood urea nitrogen
Ce: cerium
D: day
EDX: energy-dispersive X-ray spectroscopy
EP: end point
H&E: hematoxylin and eosin
ICP-MS: inductively coupled plasma mass spectrometry
ICP-OES: inductively coupled plasma optical emission spectroscopy
IF: immunofluorescence
IFN-γ: interferon gamma
IHC: immunohistochemistry
IL-1β: interleukin 1 beta
IL-2: interleukin 2
IL-6: interleukin 6
IL-10: interleukin 10
MCP-1/CCL2: monocyte chemoattractant protein-1
NP: bioglass/ceria nanoparticle
O_2_: oxygen
POD: postoperative day
ROI: region of interest
S.C.: subcutaneously
SEM: scanning electron microscopy
THF: tetrahydrofuran
TNF-α: tumor necrosis factor alpha
VEGF-A: vascular endothelial growth factor A
