# Supplementary material for: Bioglass/Ceria Nanoparticle Hybrids for the Prophylactic Treatment of Seroma: A Comparative Short-Term Study in Rats
Source: ACS Pharmacol Transl Sci. 2025 Aug 20;8(9):3170–81. doi: 10.1021/acsptsci.5c00327 (PMC12441835; doi:10.1021/acsptsci.5c00327)
Supplement: Supplementary file 1 [file pt5c00327_si_001.pdf]

SUPPORTING INFORMATION

Bioglass/ceria nanoparticle hybrids for the prophylactic treatment of seroma: a comparative short-term study in rats

Michael-Alexander Pais[1,2*], Simone de Brot[6], Robert Nißler[3,4,5], Isabel Arenas Hoyos[1,2], Athanasios Papanikolaou[1,2], Davide Bottone[3], Alexander Gogos[3], Anja Helmer[2], Robert Rieben[2], Mihai Constantinescu[1,2], Tino Matter[3,4], Inge Herrmann[3,4,5], Ioana Lese[1,2]

[1]Department of Plastic and Hand Surgery, Inselspital, University Hospital Bern, Bern 3010, Switzerland

[2]Department for BioMedical Research, University of Bern, Bern 3008, Switzerland

[3]Department of Materials Meet Life, Swiss Federal Laboratories for Materials Science and Technology (Empa), St. Gallen 9014, Switzerland

[4]Department of Mechanical and Process Engineering, ETH Zurich, Zurich 8092, Switzerland

[5]Ingenuity Lab, University Hospital Balgrist and University of Zurich 8008, Zurich, Switzerland

[6]COMPATH, Institute of Animal Pathology, University of Bern, Bern 3012, Switzerland

*** Correspondence:**

Michael-Alexander Pais

michaelalexander.pais@gmail.com

Table of Contents

Supplemental Figure 1 3
Supplemental Figure 2 4
Supplemental Figure 3 5

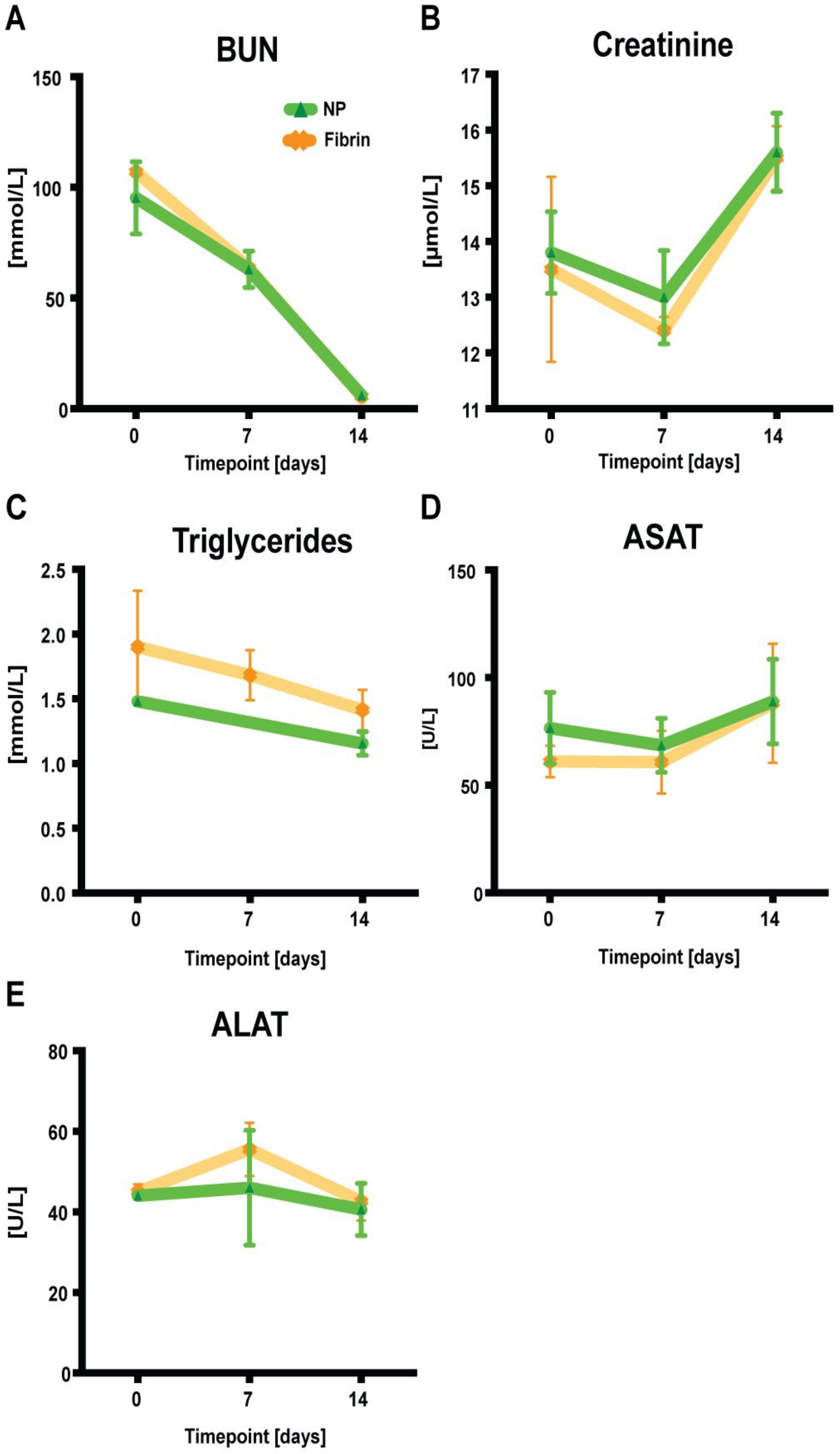

Figure S1. Plasma levels of organ-damage markers (**A-E**): NP treatment versus fibrin-glue treatment. Blood plasma assessments of BUN, creatinine, triglycerides, ASAT, and ALAT levels were made at defined timepoints. Data = mean ± SEM. Kruskal-Wallis tests with Dunn`s post-hoc for multiple comparisons indicated no significant differences between the groups.

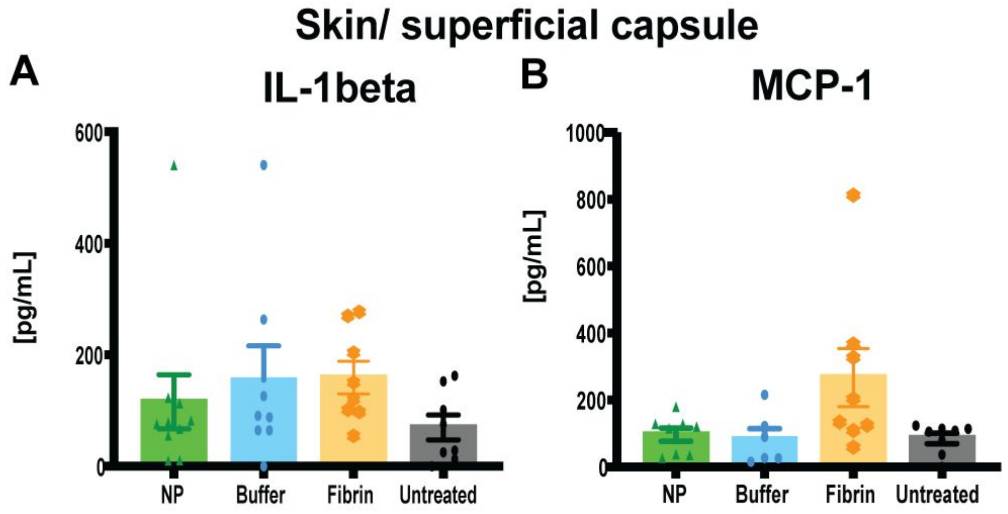

Figure S2. Biochemical analyses of IL-1beta, and MCP-1 in skin/superficial capsule (**A-B**) harvested at endpoint (POD 14). When pg/ml values were not detected (<9.45 for IL-1beta, and <14.7 for MCP-1) they were given a bin value of 0. Data = mean ± standard error of the mean. Kruskal-Wallis tests with Dunn`s post-hoc for multiple comparisons showed no significant differences between groups.

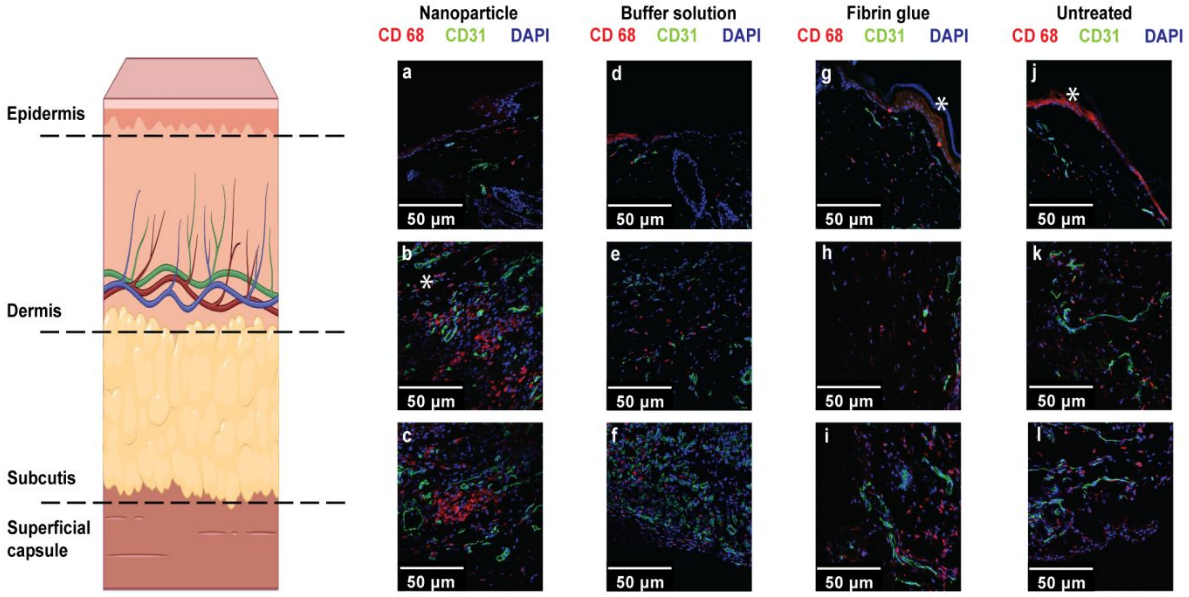

Figure S3. Confocal microscope imaging of IF staining for macrophages (CD68), and endothelial cells (CD31), with DAPI nuclear staining in skin/capsule tissue of NP treated (a-c), buffer treated (d-f), Fibrin treated (g-i) and untreated groups (j-l). Single position pictures of epidermal, intradermal and basal structures (scale bars, 50 µm). Least extensive staining intensity for CD68+ macrophages (co-localized with nuclei) was mostly observed within skin basal regions after NP and buffer solution treatment. Increased staining intensity for CD68+ after fibrin glue, and to a lesser extent, in the untreated group. Non-specific staining (*) in the epidermis and hair follicles was also observed.
